# High-Intensity Training Represses FXYD5 and Glycosylates Na,K-ATPase in Type II Muscle Fibres, Which Are Linked with Improved Muscle K^+^ Handling and Performance

**DOI:** 10.3390/ijms24065587

**Published:** 2023-03-15

**Authors:** Morten Hostrup, Anders Krogh Lemminger, Laura Bachmann Thomsen, Amanda Schaufuss, Tobias Langballe Alsøe, Gustav Krogh Bergen, Annika Birring Bell, Jens Bangsbo, Martin Thomassen

**Affiliations:** The August Krogh Section for Human Physiology, Department of Nutrition, Exercise and Sports, University of Copenhagen, 1165 Copenhagen, Denmark

**Keywords:** NKA, Na-K, ATPase, pump, FXYD, phospholemman, dysadherin, beta, subunit, isoform

## Abstract

Na^+^/K^+^ ATPase (NKA) comprises several subunits to provide isozyme heterogeneity in a tissue-specific manner. An abundance of NKA α, β, and FXYD1 subunits is well-described in human skeletal muscle, but not much is known about FXYD5 (dysadherin), a regulator of NKA and β1 subunit glycosylation, especially with regard to fibre-type specificity and influence of sex and exercise training. Here, we investigated muscle fibre-type specific adaptations in FXYD5 and glycosylated NKAβ1 to high-intensity interval training (HIIT), as well as sex differences in FXYD5 abundance. In nine young males (23.8 ± 2.5 years of age) (mean ± SD), 3 weekly sessions of HIIT for 6 weeks enhanced muscle endurance (220 ± 102 vs. 119 ± 99 s, *p* < 0.01) and lowered leg K^+^ release during intense knee-extensor exercise (0.5 ± 0.8 vs. 1.0 ± 0.8 mmol·min^–1^, *p* < 0.01) while also increasing cumulated leg K^+^ reuptake 0–3 min into recovery (2.1 ± 1.5 vs. 0.3 ± 0.9 mmol, *p* < 0.01). In type IIa muscle fibres, HIIT lowered FXYD5 abundance (*p* < 0.01) and increased the relative distribution of glycosylated NKAβ1 (*p* < 0.05). FXYD5 abundance in type IIa muscle fibres correlated inversely with the maximal oxygen consumption (*r* = –0.53, *p* < 0.05). NKAα2 and β1 subunit abundances did not change with HIIT. In muscle fibres from 30 trained males and females, we observed no sex (*p* = 0.87) or fibre type differences (*p* = 0.44) in FXYD5 abundance. Thus, HIIT downregulates FXYD5 and increases the distribution of glycosylated NKAβ1 in type IIa muscle fibres, which is likely independent of a change in the number of NKA complexes. These adaptations may contribute to counter exercise-related K^+^ shifts and enhance muscle performance during intense exercise.

## 1. Introduction

Na^+^/K^+^ ATPase (NKA) plays a vital role in muscle biology because it regulates the excitability of muscle fibres and counters the run-down of ion gradients for Na^+^ and K^+^ during muscle contractions [1,2,3]. It comprises an α and β subunit with several isoforms to form multiple isozyme complexes in a tissue-specific manner [4,5,6]. In skeletal muscle, NKA complexes reside mainly with a catalytic α subunit (α1-4) and a structural glycosylated β subunit (β1-3) [3,7,8,9]. Proteins belonging to the FXYD family anchor and regulate NKA, of which FXYD1, also known as phospholemman, is the most well described and prevalent in skeletal muscle [10,11,12,13]. However, several other members of the FXYD family exist, possibly adding further isozyme heterogeneity and specificity between muscle fibres [3,14].

FXYD5 is a dysadherin-related single-pass type I membrane glycoprotein that regulates NKA. While mainly expressed in epithelial tissues and markedly upregulated in carcinomas [15,16], FXYD5 is also expressed in skeletal muscle [15,16,17,18]. However, not much is known about FXYD5 in human skeletal muscle. Persons with spinal cord injuries express muscle FXYD5 at >10-fold higher levels than healthy comparators [18], which implies that muscle activation regulates FXYD5 expression. Thus, it is likely that FYXD5 expression in skeletal muscle is exercise-responsive, similar to the FXYD1 auxiliary protein [19,20].

Human skeletal muscle tissue is heterogeneous. It consists of muscle fibres with different functional and metabolic characteristics [21], which typically are classified as slow- or fast-twitch, depending on their expression of myosin heavy chain (MHC) isoforms. Given that muscle tissue also comprises other cell types, including endothelial, inflammatory, and neural cells, as well as connective tissue, emphasises the need for a refined single muscle fibre analysis to provide a homogenous and cleaner portrayal of muscle fibre characteristics [12,21]. NKA subunits, including FXYD1, demonstrate some muscle fibre-type specificity, are influenced by sex, and exhibit remarkable adaptability to exercise training and inactivity [3,22,23,24,25].

The putative role of FXYD5 in the regulation of NKA and, hence, K^+^ shifts during muscle contractions is unknown in humans. Boon et al. (2012) speculated that FXYD5 upregulation in spinal cord injury patients could be a compensatory mechanism to facilitate NKA activity, as these patients had low NKA expression. However, in cell models, FXYD5 decreases the degree of glycosylation of NKAβ1 and destabilises the NKA complex [15,16,26]. Therefore, it is possible that exercise training leads to a greater distribution of glycosylated NKAβ1 in skeletal muscle. This calls for an in-depth investigation of the muscle fibre type-specific responsiveness of FXYD5 to a period of exercise training and its putative role in glycosylated NKAβ1 and K^+^ regulation of contracting muscles during exercise in humans.

Thus, we examined muscle fibre type-specific adaptations in FXYD5 and glycosylated NKAβ1 in response to a period of high-intensity interval training (HIIT) in young males. Since spinal cord injury markedly increases muscle FXYD5 content, we hypothesised that HIIT would lower muscle FXYD5 abundance and consequently increase the relative distribution of glycosylated NKAβ1. Furthermore, we investigated the effect of HIIT on the abundance of NKAα2 and β1, in addition to functional measures of K^+^ regulation and performance during isolated muscle exercises. Last, we examined sex and muscle fibre-type differences in FXYD5 abundance.

## 2. Results

### 2.1. HIIT Counters Exercise-Related K^+^ Shifts and Enhances Muscle Performance

Nine habitually active young males performed a 6-week HIIT intervention comprising 3 weekly sessions of 1-min all-out intervals on indoor spinning bikes interspersed by 2-min active recovery repeated 5 times during the first week and progressively increasing to 10. Subjects were 23.8 ± 2.5 years (mean ± SD), had a VO_2max_ of 47 ± 7 mL⋅min^–1^⋅kg^–1^, and a BMI of 23.3 ± 1.4 kg⋅m^–2^. 

**Figure 1:**
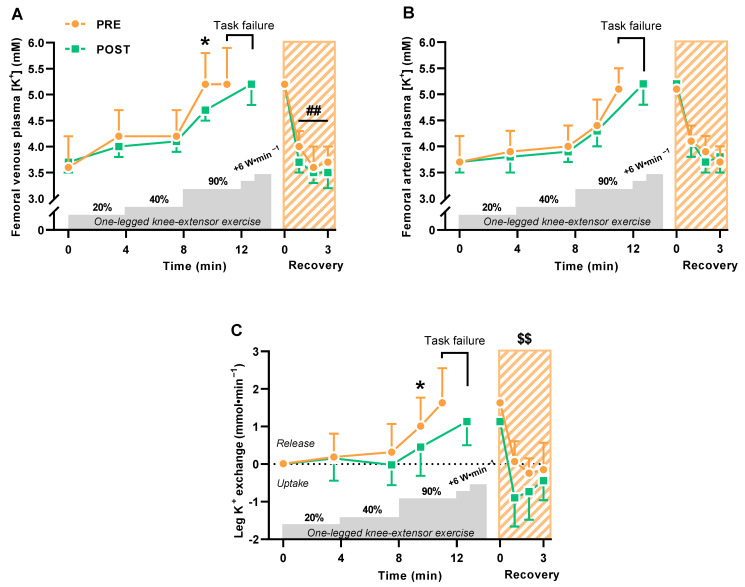
Exercise-related K^+^ shifts during and 0–3 min into recovery from one-legged knee extensor exercise before (PRE) and after (POST) 6 weeks of high-intensity interval training (HIIT) 3 times weekly in young males (n = 9). (**A**,**B**) Femoral venous (**A**) and arterial (**B**) plasma K^+^ concentrations. (**C**) Leg K^+^ release and uptake. Data are presented as mean ± SD. * POST different from PRE (*p* < 0.05). ^##^ POST recovery values different from PRE (*p* < 0.01). ^$$^ Cumulated leg K^+^ uptake after HIIT different from before (*p* < 0.01).

The 6-week period with HIIT lowered (*p* = 0.023) femoral venous plasma K^+^ concentrations during knee extensor exercise at high intensity but not significantly at low and moderate intensity (Figure 1A). Femoral venous plasma K^+^ concentrations reached similar levels at task failure from high-intensity incremental exercise before and after HIIT, but task failure incurred much later after HIIT (220 ± 102 vs. 119 ± 99 s, *p* < 0.0001) (Figure 1A). In the immediate recovery from exercise 0–3 min after task failure, femoral venous plasma K^+^ concentrations declined (*p* = 0.006) more after HIIT than before (Figure 1A). Femoral arterial plasma K^+^ concentrations did not change with HIIT (Figure 1B).

Leg K^+^ release rate was lower during exercise at high intensity after HIIT than before (0.5 ± 0.8 vs. 1.0 ± 0.8 mmol·min^–1^, *p* = 0.008) (Figure 1C), while cumulated leg K^+^ reuptake 0–3 min into recovery was greater after HIIT than before (2.1 ± 1.5 vs. 0.3 ± 0.9 mmol, *p* = 0.007) (Figure 1C).

### 2.2. HIIT Downregulates Muscle FXYD5 Independent of Changes in NKA Isoforms

**Figure 2:**
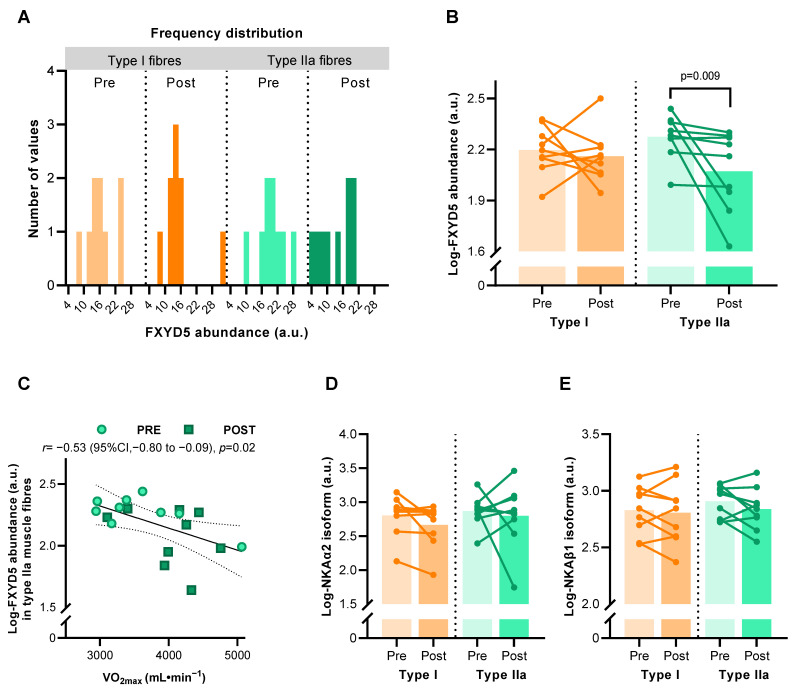
Abundance of Na, K-ATPase (NKA) subunits in human muscle type I and IIa fibre pools before (Pre) and after (Post) 6 weeks of high-intensity interval training (HIIT) 3 times weekly in young males (*n* = 8–9). (**A**) Frequency distribution of FXYD5 abundance in human type I and IIa muscle fibres before log-transformation. (**B**) Effect of HIIT on log-FXYD5 abundance. (**C**) Pearson’s correlation between log-FXYD5 abundance and maximal oxygen consumption (VO_2max_). (**D**,**E**) Effect of HIIT on log-NKAα2 (**D**) and log-NKAβ1 abundance (**E**). Bars represent mean values.

A total of 630 single muscle fibres from nine males who completed the HIIT intervention revealed some inter-subject variability that partially violated normality (for type IIa fibres after HIIT) (Figure 2A). Mixed model analysis on log-transformed FXYD5 abundance values showed an effect of HIIT, which was driven by a reduction of FXYD5 abundance in type IIa muscle fibres (*p* = 0.009) but not in type I fibres (Figure 2B). Pearson correlation coefficient analysis showed that the muscle log-FXYD5 abundance correlated with maximal oxygen consumption (VO_2max_) (*r* = –0.532, *p* = 0.023) in type II fibres only (Figure 2C). NKAα2 and β1 subunit abundance did not change significantly with HIIT in either fibre type (Figure 2D,E).

### 2.3. HIIT Increases Muscle NKAβ1 Glycosylation

**Figure 3:**
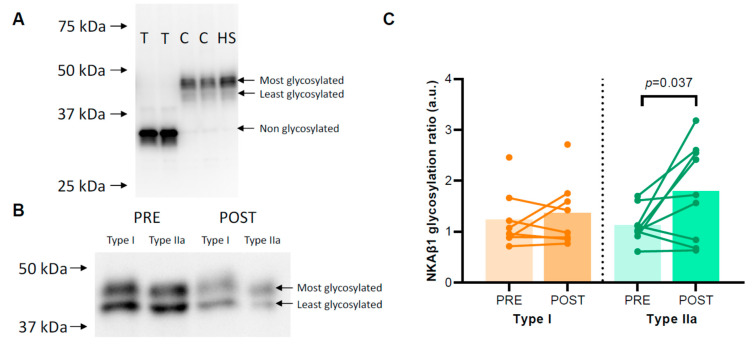
Degree of glycosylation of the Na, K-ATPase (NKA) β1 subunit. (**A**) A control experiment indicating that the differences between the two upper signals migrating between 40 and 50 kDa are related to the degree of NKAβ1 glycosylation. Human skeletal muscle samples treated with 1 unit N-glycosidase per 40 µg protein (T) or controls without N-glycosidase (C) and a human skeletal muscle lysate (HS) were loaded on a gel, and afterwards, the membrane was incubated with an NKAβ1 antibody. (**B**) Representative Western blots for the pooled single fibres are shown. The NKAβ1 glycosylation ratio was determined as the ratio between the most glycosylated signal and the least glycosylated signal between 40 and 50 kDa. (**C**) NKAβ1 glycosylation ratios in human skeletal muscle type I and IIa fibres of healthy young men (*n* = 8–9) before (PRE) and after (POST) 6 weeks of high-intensity interval training (HIIT) 3 times weekly.

The degree of NKAβ1 glycosylation was not different between type I and type IIa muscle fibres (1.45 ± 0.22 vs. 1.46 ± 0.18, *p* = 0.946). HIIT raised the NKAβ1 glycosylation ratio in type IIa muscle fibres from 1.12 ± 0.11 before to 1.80 ± 0.30 after HIIT (*p* = 0.037) but not in type I muscle fibres (*p* = 0.206) (Figure 3B). For type IIa muscle fibres, Spearman’s rho revealed a significant negative correlation between FXYD5 abundance and NKAβ1 glycosylation ratio (*r* = −0.577, *p* = 0.012), whereas no such relation was apparent in type I fibres (*r* = −0.188, *p* = 0.485).

### 2.4. Fibre Type-Specific FXYD5 Abundance and Influence of Sex

**Figure 4:**
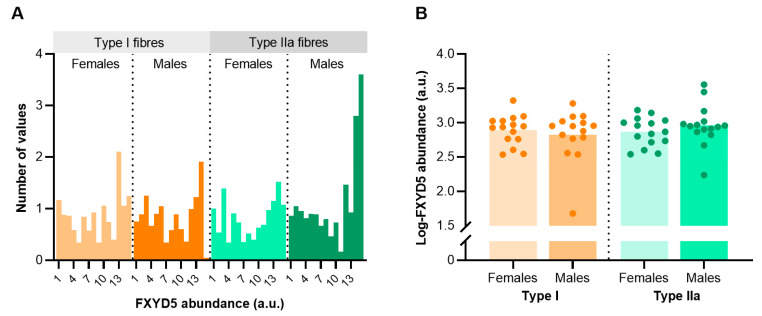
FXYD5 abundance in human skeletal muscle type I and IIa fibres of healthy young females (*n* = 15) and males (*n* = 15). (**A**) Frequency distribution of FXYD5 abundance before log-transformation. (**B**) Individual data points for both sexes and fibre types. Bars represent sample means.

To determine sex- and fibre type-specific differences in FXYD5 abundance, we dissected and pooled around 1050 single fibres of biobank vastus lateralis biopsies obtained from 30 trained males and females (*n* = 15 from each sex) matched for age (21–35 years). The analysis revealed some inter-subject variability and skewness for FXYD5 abundance in both type I and IIa fibres that violated normality (Figure 4A). Thus, we used log-transformed FXYD5 data for subsequent comparative statistical analyses. Mixed model analysis showed no sex differences in FXYD5 abundance between trained males and females (*p* = 0.872). Furthermore, the analysis revealed no apparent fibre type differences, though the abundance was numerically, but non-significantly, higher in type IIa fibres than type I fibres in males (*p* = 0.181) (Figure 4B).

## 3. Discussion

The key findings of this study were that HIIT downregulated FXYD5 and increased the relative distribution of glycosylated NKAβ1 in type IIa muscle fibres of young males. These adaptations occurred independent of changes in NKAα2 and NKAβ1 abundance and were associated with improved K^+^ regulation and muscle performance during intense knee extensor exercise. Thus, our findings highlight FXYD5 as a potential player in the complex regulation of NKA and K^+^ shifts during muscle contractions.

The finding that HIIT downregulated FXYD5 agreed with our working hypothesis. While this effect only was evident in type IIa muscle fibres, the finding extends to the opposite phenomenon observed in patients with spinal cord injury, in which FXYD5 abundance in mixed muscle homogenates is markedly upregulated [18]. This implies that muscle activity and innervation regulate FXYD5 expression in human muscle fibres. However, electrical pulse stimulation of human cultured myotubes for 48 h, as a model of chronic muscle activity, upregulated FXYD5 abundance, while innervation did not affect its abundance [17]. Discrepancies between the in situ and in vitro findings may reflect that other factors also regulate FXYD5 in muscle fibres, including the metabolic and hormonal milieu. HIIT induces pronounced muscle metabolic disturbances and hormonal fluctuations [27,28,29,30] that are difficult to replicate in cells [31]. Several circulating hormones have also been shown to affect the expression of NKA. For example, adrenal hormones cortisol (glucocorticoids), aldosterone, and epinephrine (and adrenergic compounds) have all been shown to regulate NKA expression in skeletal muscle [1,32,33,34].

The downregulation of FXYD5 with HIIT occurred despite no concomitant changes in NKAα2 and β1 abundance, which indicates that the repression of FXYD5 arose independently of a change in the number of NKA complexes. This is similar to FXYD1, which can exhibit a fibre type-specific responsiveness to exercise training independent of changes in NKAα and β subunits [35]. The lack of change in NKAα2 and β1 abundance, irrespective of fibre type, was nevertheless surprising, as HIIT generally upregulates NKA isoforms [22,36,37,38,39]. However, not all HIIT studies found an effect on NKAα2 and β1 abundance [40,41,42], and studies have demonstrated substantial fibre type specificity in the responsiveness of NKA isoforms to exercise training [24,43,44]. This emphasises the importance of performing fibre type-specific analyses when determining NKA subunits and FXYD adaptability to exercise training, as demonstrated in this and other studies [3,45].

The implications of a lower abundance of FXYD5 with HIIT likely means that membrane-bound NKA complexes will be under less FXYD5-mediated deglycosylation of NKAβ1 [15,16,26]. We observed that HIIT not only increased the relative distribution of glycosylated NKAβ1 in type IIa muscle fibres but also that the degree of glycosylated NKAβ1 correlated inversely with FXYD5 abundance. Given that a greater distribution of glycosylated NKAβ1 may stabilise NKA complexes [15,16,26], such an adaptation could be of importance during intense muscle activity, where metabolic perturbations and redox disturbances compromise NKA function [3]. Indicative of improved NKA function, we observed that HIIT enhanced leg muscle performance and countered exercise-related K^+^ shifts, as reflected by a lower leg K^+^ release and femoral venous K^+^ accumulation during intense exercise and greater leg K^+^ reuptake during recovery. Furthermore, FXYD5 abundance in type IIa muscle fibres correlated inversely with VO_2max_. This collectively implies that FXYD5 contributes to the complex regulation of NKA and may have a role in K^+^ regulation in skeletal muscle during exercise and, hence, influence ionic disturbances and fatigue development.

Similar to that of FXYD1 [12], we observed no apparent differences between the fibre types in FXYD5 abundance. However, human muscle fibre-type differences in NKA isoforms have been shown with some inconsistency. β2 subunit expression, in particular, appears fibre type-specific, with a higher abundance in type II muscle fibres than in type I of young and aged individuals [23,24,35], whereas this does not seems to be the case for the β1 subunit [12,23]. For NKAα2, its abundance was greater in type II muscle fibres than in type I fibres of recreationally active males in some [12,35], but not all, studies [23,24]. Thus, muscle fibre-type differences appear isoform-specific in humans and are not apparently evident for the auxiliary members of the FXYD family.

We also observed no sex differences in FXYD5 abundance for either muscle fibre type. Murphy et al. (2007) observed a higher muscle mRNA expression of the NKAα3 and β3 subunits for recreationally active males than females but not for either the total NKA content or maximally stimulated activity. A putative role of sex hormones, testosterone, and oestrogen in the regulation of NKA abundance has been shown in rat soleus muscle [46] and cardiomyocytes [47], respectively. Furthermore, the oestrogen receptor has been implicated in the regulation of dysadherin in multiple large-scale studies on cancer progression [48]. Hence, it is likely that sex hormones play some part in the expression of NKA isoforms in human skeletal muscle but not clearly for FXYD5—at least not in young, trained individuals.

The beneficial health and performance-related effects of exercise are indisputable [49,50]. The bulk of these effects lie in the exceptional exercise-responsiveness of skeletal muscle [21,51,52]. While it is well known that NKA isoforms and FXYD1 are among the most exercise-adaptive proteins and contribute to enhancing muscle fatigue resilience and performance [3], the present study extends previous studies on the regulation of NKA [3,22] by demonstrating that FXYD5 may also be a part of the complex exercise-related regulation of NKA. A limitation of our longitudinal training study was the small sample size. However, almost all the males undergoing the training intervention had a variable repression of FXYD5 abundance in type IIa fibres (eight out of nine). Furthermore, the three subjects who had a particularly large reduction in FXYD5 abundance with training had a concomitantly large increase in the relative distribution of glycosylated NKAβ1.

In summary, our findings demonstrate that FXYD5 exhibits muscle fibre type-specific adaptability to HIIT performed at the near-maximal intensity in young males. While FXYD5 abundance in type I muscle fibres was unrelated to the fitness level and did not respond to HIIT, its abundance in type IIa muscle fibres was associated with fitness level and declined in response to 6 weeks of HIIT. The downregulation of FXYD5 in type IIa muscle fibres was associated with a greater relative distribution of glycosylated NKAβ1 after HIIT. These adaptations were likely independent of a change in the number of NKA complexes, as NKAα2 and β1 abundance did not change with HIIT and was associated with an enhanced muscle endurance and lower muscle K^+^ release and accumulation of femoral venous K^+^ during intense exercise. Collectively, this highlights FXYD5 as a possible exercise-responsive regulator of the degree of NKAβ1 glycosylation that contributes to the complex regulation of NKA in skeletal muscle and, hence, K^+^ shifts during muscle contractions in humans.

## 4. Materials and Methods

### 4.1. Subjects

Nine habitually active, but otherwise untrained, young males participated in this longitudinal training intervention study comprising 6 weeks of HIIT. Before inclusion, subjects received oral and written information about the contents and risks of the study, and each subject gave oral and written informed consent. Inclusion criteria were healthy males, aged 18–40 years, maximal oxygen consumption (VO_2max_) 45–55 mL⋅min^–1^⋅kg^–1^, and BMI 19–26 kg⋅m^–2^. Exclusion criteria were abnormal electrocardiogram, chronic disease, ongoing medical treatment, and smoking. The study was approved by the Committee on Health Research Ethics of the Capital region (H-17004045) and conducted in accordance with the Declaration of Helsinki (2013). The study was registered at clinicaltrials.gov (NCT03317704).

### 4.2. Assessment of Eligibility and Familiarisation

For the assessment of eligibility, subjects underwent a medical examination consisting of a health questionnaire about previous events and medicine use, as well as a heart and lung auscultation, followed by an electrocardiogram. Then, subjects completed an incremental test to exhaustion on a bike ergometer (Monark LC7TT, Monark Exercise, Vansbro, Sweden) to determine VO_2max_ and exercise capacity. Following a short break, subjects were familiarised with one-legged knee extensor exercise and were instructed in contracting the quadriceps while relaxing the thigh during knee flexion and maintaining a cadence of 60 RPM.

At least two days after the screening, subjects returned to the laboratory for a familiarisation visit, where subjects performed a one-legged knee extensor incremental test to task failure for determination of incremental peak power output of the leg (W_max_). The protocol started at 12 W and increased by 6 W⋅min^–1^ until task failure, which was defined as an inability to maintain a cadence of 60 RPM for 10 s or a drop-in cadence below 55 RPM, despite strong verbal encouragement. W_max_ was calculated from task failure in a time-dependent manner, considering the amount of time spent on the last increment. Leg W_max_ of the subjects was 61 ± 14 W.

### 4.3. Pre- and Post-Training Intervention Trials

Before and after the 6-week training intervention comprising HIIT 3 times weekly, subjects underwent an experimental trial. Subjects met at the laboratory in the morning after an overnight fast and rested in a supine position for 10 min. Hereafter, subjects received a standardised meal with 500 mL water and rested for two hours. During this period, we inserted catheters (20 gauge, Teleflex, Wayne, PA, USA) into the femoral artery and vein, under local anaesthesia (5 mL Xylocaine^®^, 20 mg⋅mL^–1^ lidocaine without adrenaline, AstraZeneca, London, UK) below the inguinal ligament and advanced the catheters proximally with ultrasound guidance (Vivid E9, GE, Healthcare, Waukesha, WI, USA). During the last 15 min of the resting period, we sampled a muscle biopsy at the belly of the vastus lateralis using a Bergström needle with suction [53]. Before biopsy sampling, we applied local anaesthesia (2 mL Xylocaine^®^). The sampled muscle specimen was quickly washed in ice-cold saline, dried, frozen in liquid nitrogen, and stored at –80 °C until analysis.

After the resting period, subjects performed one-legged knee extensor exercise at 60 RPM for 4 min at low intensity (20% leg W_max_), 4 min at moderate intensity (40% leg W_max_), and 4 min at high intensity (90% leg W_max_), followed by increments of 6 W⋅min^–1^ until task failure as defined above. We collected femoral arterial and venous blood before and during the last minute of each workload until task failure, as well as 1, 2, and 3 min into recovery. Venous blood was sampled ≈6 s after arterial blood to account for the mean transit time of arterial blood through the capillary bed [54]. A trained technician measured the femoral arterial blood flow at the blood sampling time points using ultrasound Doppler (Vivid E9, GE, Healthcare, Waukesha, WI, USA).

The post-intervention experimental trial was conducted 3–4 days after the final training session. Subjects were told to refrain from exercise 48 h before the experimental days and caffeine and alcohol 24 h before the experimental days. In addition, subjects recorded their food and fluid intake 24 h before the pre-intervention experimental trial, which was replicated the day before the post-intervention experimental trial.

#### Training Intervention

During the 6-week training intervention, subjects performed 3 weekly sessions of HIIT on indoor spinning bikes. Training sessions consisted of a 10-min warm-up with two 5-s sprints, followed by 1-min all-out intervals interspersed by 2-min active recovery repeated 5 times during the first week and progressively increasing to 10 intervals during the final week. This type of HIIT effectively augments the capacity for ion transport in skeletal muscle and enhances the ability to counter exercise-related K^+^ shifts [3,22]. The training sessions were supervised by instructors who provided verbal encouragement during each interval. All subjects had 100% training compliance.

### 4.4. Experimental Procedures

#### 4.4.1. Maximal Oxygen Consumption during Incremental Bike Ergometer Exercise

The VO_2max_-test protocol consisted of a 4-min period at 100 W followed by increments in the workload of 25 W⋅min^–1^ until exhaustion on a bike ergometer. During the test, pulmonary gas exchange was measured breath-by-breath using an online gas analyser (Oxycon Pro, CareFusion, Hoechberg, Germany). VO_2max_ was determined as the highest value reached over a 30-s period. At least two of the following criteria had to be met before the test was approved: a plateau in oxygen uptake despite an increase in workload, a respiratory exchange ratio of above 1.15, or inability to maintain a cadence above 80 RPM for 5 s, despite strong verbal encouragement.

#### 4.4.2. Femoral Arterial and Venous Blood Samples and Blood Flow

Femoral arterial and venous blood samples were drawn in heparinised syringes and immediately analysed with a blood gas analyser (ABL800 FLEX, Radiometer, Copenhagen, Denmark) for plasma K^+^ concentration, haemoglobin, and haematocrit. Femoral arterial blood flow was measured using Doppler ultrasonography (Vivid E9) with a linear probe operating at an image frequency of 8.0 MHz and a Doppler frequency of 3.1 MHz, as previously described [55]. Blood flow was measured over a 15-s period before and after each blood sampling, with the average of these blood flows being used for data analysis.

#### 4.4.3. Leg Plasma K^+^ Shifts

The net plasma K^+^ exchange (release or uptake) of the leg was calculated as previously described [56,57], accounting for net transcapillary water exchange (*J_v_*) into or out of the vein:

$${Exchange}_{ion}=\left(F_{p}-J_{v}\right)\cdot {ion}_{v}-F_{p}\cdot {ion}_{a}$$

where *ion_v_* and *ion_a_* are the venous and arterial ion concentrations, respectively.

Femoral arterial plasma flow (*F_p_*) was calculated as:

$$F_{p}=F\cdot \left(1-\frac{{Hct}_{a}}{100}\right)$$

where *F* is the femoral arterial blood flow, and *Hct_a_* is the arterial haematocrit.

*J_v_* was calculated as:

$$J_{v}=F\cdot \left(\left[\left(\frac{{Hb}_{a}}{{Hb}_{v}}\right)\cdot \left(\frac{100-{Hct}_{v}}{100-{Hct}_{a}}\right)\right]-1\right)$$

where *Hb_a_* and *Hb_v_* are the arterial and venous haemoglobin concentrations, respectively, and *Hct_v_* is the venous haematocrit.

#### 4.4.4. Human Muscle Single Fibre Dissection

We used a microscope and fine forceps (Fine Science Tools GmbH, Heidelberg, Germany) to dissect sections of single human muscle fibres from freeze-dried biopsy samples as described previously [21,32]. Around 35 single fibre pieces 1–2 mm in length were collected from each muscle sample and placed in the bottom of a 0.5 mL tube and spun at 2000× *g* using a table centrifuge (Kinetic Energy 26 Joules Galaxy Mini Centrifuge, VWR, Søborg, Denmark) before each single fibre piece was dissolved in 10 µL of 3 × sample buffer (6 × Laemmli buffer: 7 mL 0.5 M Tris base, 3 mL glycerol, 0.93 g DTT, 1 g SDS, and 1.2 mg bromophenol blue diluted 1:1 with 0.5 M Tris base) and vortexed thoroughly. After another short table centrifugation step, the fibre type was determined by dot blotting, as described below.

#### 4.4.5. Fibre Typing Using Dot Blotting

We determined muscle fibre types using dot blotting, as previously described [58]. We activated PVDF membranes in 96% ethanol for 15–30 s and then equilibrated them for 5 min in transfer buffer (0.58% Tris base, 0.29% glycine, 0.015% SDS, and 20% ethanol). The wet membranes were then placed on a stack of filter papers, where two wet pieces soaked in transfer buffer were on top of three dry pieces. We next aliquoted 1 µL, corresponding to 1/10 of the dissected fibre segment, and spotted it on two separate membranes. Samples obtained from the same biopsy were spotted on the same two membranes. After complete absorption of the samples, we moved the membranes to a filter paper to dry for ≈5 min. The dried membranes were reactivated in 96% ethanol for 15–30 s and equilibrated in transfer buffer for 5 min. After three quick washes in Tris-based saline with 0.1% Tween-20 (TBST), we blocked the membranes with 2% skimmed milk in TBST for 5 min at room temperature on a rocking table. We then rinsed the membranes with TBST before incubation of the two identical membranes with either an MHCI (A4.840) or MHCIIa (A4.74) antibody (diluted 1:200 in 3% BSA in TBST, Developmental Studies Hybridoma Bank, University of Iowa, USA) for 2 h gently rocking at room temperature. After incubation in the primary antibody, we washed the membranes in TBST before a 1-h incubation with an HRP-conjugated goat anti-mouse secondary antibody (1:5000 in 2% skimmed milk TBST, P-0447 Dako, Glostrup, Denmark) at room temperature on a rocking table. Membranes were then washed three times in TBST and exposed to an enhanced chemiluminescence (ECL) reagent (Immobilon Forte Western HRP substrate, Merck Millipore, Darmstadt, Germany) and imaged (ChemiDoc MP Imaging System, Bio-Rad, Hercules, CA, USA).

**Figure 5:**
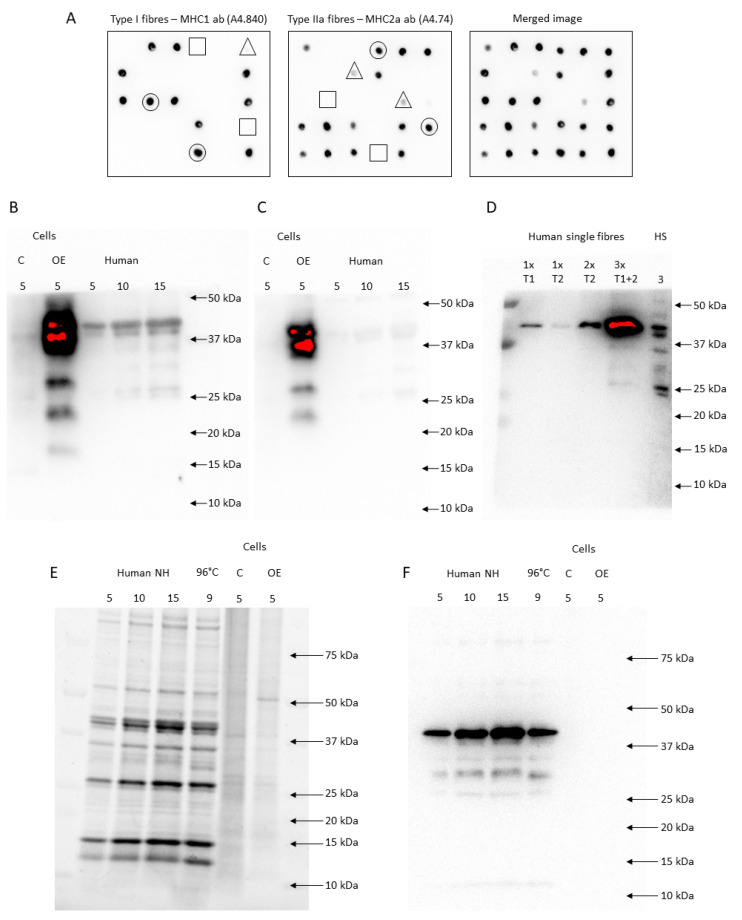
Dot blotting and FXYD5 antibody validation (HPA010817 and SC-166782). (**A**) Shows dot blotting of 6 × 5 fibre segments incubated with either an MHC1 or MHC2a antibody. Each dot was evaluated as no signal (□), a weak signal (∆), or a clear signal (○). (**B**,**C**) HPA010817 (**B**) and SC-166782 (**C**) antibodies showed a clear multiple-band signal (19–40 kDa and 22–40 kDa, respectively) in FXYD5 overexpression samples (OE) compared to the control (C) and human muscle lysates. (**D**) In the first two lanes, one segment of type I (T1) and type IIa fibre (T2), respectively, was loaded. In the third lane, two pooled type IIa fibres were loaded, while pooled segments from three different fibres were loaded in lane four. In lane five, 3 µg human skeletal muscle lysate was loaded. The membrane was incubated with HPA010817. (**E**) The stain-free image is shown from loaded human skeletal muscle lysates either non-heated (NH) or heated for 3 min at 96 °C (96 °C) and FXYD5 OE and C samples. (**F**) Shows the membrane after incubation of the samples from (**E**) with an anti-actin antibody.

We determined each sample to be a type I or type IIa fibre based on signal intensities. Only fibre segments with a clear signal concomitant and no signal in the opposite staining were included in further analyses (Figure 5A). Hence, hybrid fibre segments (signals in both stainings) and potential type IIx fibres (no signal in both stainings) were not used for further analyses. We then pooled all type I and type IIa fibres for each subject and time point.

#### 4.4.6. Immunoblotting and SDS Page

We performed immunoblotting on muscle fibre pools, as previously described [59]. We loaded an equal amount of the pooled type I and type IIa fibres from the same subject on the same gel (4–15% TGX Stain-Free^TM^, Bio-Rad), together with two protein markers (Precision plus all blue, Bio-Rad) and three human skeletal muscle standard samples, obtained as a pool of samples. After SDS-page gel electrophoresis, proteins were transferred semi-dry to a PVDF membrane. The total protein amount loaded in each lane was determined as the stain-free signal by 5 min of UV light incubation of the gels before a digital picture was obtained (ChemiDoc MP Imaging System, Bio-Rad, Hercules, CA, USA). The same part of the gels (5–50 kDa for FXYD5 analyses) was placed on one membrane. The empty parts on the membrane were blocked with either 2% skimmed milk or 3% BSA in TBST before overnight incubation with a primary antibody. The following antibodies were used with the migration of the quantified signal noted: NKAα2: 100 kDa, 07-647 (Merck Millipore, Darmstadt, Germany); NKAβ1: 40–50 kDa, MA3-930 (Affinity Bioreagents, Golden, CO, USA); and FXYD5: 19–40 kDa, HPA010817 (Merck Sigma-Aldrich, Darmstadt, Germany) (see validation below). The membrane was washed in TBST, incubated for 1 h in HRP-conjugated secondary antibody (goat anti-mouse: P-0447 DAKO, Glostrup, Denmark and goat anti-rabbit: 4010-05 (Southern Biotech, Birmingham AL, USA) at room temperature and washed 3 × 15 min in TBST before the bands were visualised with an ECL reaction and signals recorded with a digital camera (Bio-Rad, Hercules, CA, USA). Densitometry quantification of the immunoblotting band intensities was done using Image Lab version 4.0 (Bio-Rad, Hercules, CA, USA) and determined as the total band intensity adjusted for the background intensity. A three-point standard curve on each gel was used to confirm that the loaded amount of samples was capable of determining differences between samples by the signal intensity being on the linear and steep part of the standard curve. Either the average of the triplicate human standard sample signal loaded across each gel or the average of all pooled single fibre samples was used for the normalisation of all samples on the gel to allow for semi-quantitative comparisons across gels and subjects.

#### 4.4.7. FXYD5 Antibody Validation and Specificity

We tested the specificity of two anti-FXYD5 antibodies (HPA010817, Merck Sigma-Aldrich and sc-166782, Santa Cruz Biotechnology, Dallas, TX, USA). Both antibodies showed multiple signals from 19 to 40 kDa in human skeletal muscle homogenates and FXYD5 overexpression (OE) cell lysates compared to the control (C) (LY408093, OriGene Technologies, Rockville, MD, USA), indicating that the abs recognise FXYD5 (Figure 5B,C). Afterwards, we tested the effect of the amount of protein loaded. With a low amount of protein loaded onto the gels, equivalent to a single muscle fibre, only the 40-kDa band was present. However, bands below 40 kDa were present when 3 pooled fibres were loaded, indicating that it is possible to detect FXYD5 in both single fibres (single band at ~40 kDa) and homogenates (multiple bands), while the amount of protein is important to visualise all bands (Figure 5D). According to the UniProt database, human FXYD5 is a 178 amino acid and 19 kDa protein (canonical sequence) with potential isoforms. However, given that FXYD5 is subjected to post-translational O-glycosylation [60,61] and due to “abnormal electrophoretic mobility” [16], it migrates with varying weights, as shown in mice (24–55 kDa) [16,60,61], Xenopus oocytes (24 kDa) [16], MIA PaCa-2 and Panc-1 cells (30–45 kDa) [62], A549 human lung cancer cells (32–55 kDa) [63], and the present study (19–40 kDa).

Since FXYD5 migrated around 40 kDa in our human muscle samples, we tested if the antibodies bound to actin. The stain-free signal indicated that the total amount of protein was similar between OE and C and that actin (~42 kDa) was not highly expressed in these samples (Figure 5E). Incubation with an anti-actin antibody (A2066, Merck Sigma-Aldrich, Darmstadt, Germany) revealed no presence of actin in FXYD5 overexpression cell lysates (OE), while it showed expected strong signals in the human skeletal muscle samples (Figure 5F). Thus, the stronger positive signals with two different commercial antibodies (19–40 kDa) in FXYD5 overexpression cell lysates than in the control samples (Figure 5B,C) are not due to binding to actin. From these ab tests, we concluded that both abs bound specifically to FXYD5 proteins in human skeletal muscle samples and used the HPA010817 antibody (Merck Sigma-Aldrich, Darmstadt, Germany) to determine the FXYD5 abundance in human skeletal muscle.

#### 4.4.8. NKAβ1 Glycosylation

NKAβ1 is glycosylated at three *N*-linked glycosylation sites, and because of this, it migrates with a double band between 40 and 50 kDa in human skeletal muscle (Figure 3A). We confirmed that NKAβ1 glycosylation caused the two-band signal in human muscle samples using a deglycosylation kit (P0753, Lambda Protein Phosphatase, New England BioLabs, Ipswich, MA, USA). We added either 1 unit of N-glycosidase per 40 µg protein or a control buffer without N-glycosidase to a human skeletal muscle lysate and incubated the lysate at 37 °C for 60 min. Total deglycosylated NKAβ1 migrated at 35 kDa, whereas the control samples and the original human muscle sample migrated at 40–50 kDa (Figure 3A). Thus, to determine the relative distribution of glycosylated NKAβ1 in the single fibre pools before and after HIIT, we calculated the ratio between the most and least glycosylated NKAβ1 subunits (upper and lower bands between 40 and 50 kDa, respectively, Figure 3B).

#### 4.4.9. Statistics

We used SPSS version 28 (IBM, Armonk, NY) for the statistical analyses. Normality was assessed using the Shapiro–Wilk test and histograms. To estimate the influence of sex and fibre type on NKAα2, NKAβ1, and FXYD5 abundance, we utilised a linear mixed model with the sex and fibre type as fixed factors and a random intercept for subjects. To estimate the effect of training on protein abundance in type I and IIa muscle fibres, we performed a repeated-measures linear mixed model with the training and fibre type as fixed factors and a random intercept for subjects. Outcome statistics are presented with exact *p*-values to represent probability. Data are presented as mean ± SD unless otherwise specified.

## Data Availability

Data are not publicly available due to GDPR regulations.

## References

[1] Clausen T. (2003). Na^+^-K^+^ pump regulation and skeletal muscle contractility. Physiol. Rev..

[2] Wyckelsma V.L., Perry B.D., Bangsbo J., McKenna M.J. (2019). Inactivity and exercise training differentially regulate abundance of Na^+^-K^+^-ATPase in human skeletal muscle. J. Appl. Physiol..

[3] Hostrup M., Cairns S.P., Bangsbo J. (2021). Muscle Ionic Shifts During Exercise: Implications for Fatigue and Exercise Performance. Compr. Physiol..

[4] Blanco G., Mercer R.W. (1998). Isozymes of the Na-K-ATPase: Heterogeneity in structure, diversity in function. Am. J. Physiol.-Ren. Physiol..

[5] Geering K. (2008). Functional roles of Na, K-ATPase subunits. Curr. Opin. Nephrol. Hypertens..

[6] Geering K. (2006). FXYD proteins: New regulators of Na-K-ATPase. Am. J. Physiol.-Ren. Physiol..

[7] Pirkmajer S., Chibalin A.V. (2016). Na, K-ATPase regulation in skeletal muscle. Am. J. Physiol.-Endocrinol. Metab..

[8] Murphy K.T., Snow R.J., Petersen A., Murphy R.M., Mollica J., Lee J.S., Garnham A.P., Aughey R., Leppik J.A., Medved I. (2004). Intense exercise up-regulates Na^+^, K^+^-ATPase isoform mRNA, but not protein expression in human skeletal muscle. J. Physiol..

[9] Benziane B., Widegren U., Pirkmajer S., Henriksson J., Stepto N.K., Chibalin A.V. (2011). Effect of exercise and training on phospholemman phosphorylation in human skeletal muscle. Am. J. Physiol.-Endocrinol. Metab..

[10] Crambert G., Füzesi M., Garty H., Karlish S., Geering K. (2002). Phospholemman (FXYD1) associates with Na, K-ATPase and regulates its transport properties. Proc. Natl. Acad. Sci. USA.

[11] Bibert S., Roy S., Schaer D., Horisberger J.-D., Geering K. (2008). Phosphorylation of phospholemman (FXYD1) by protein kinases A and C modulates distinct Na, K-ATPase isozymes. J. Biol. Chem..

[12] Thomassen M., Murphy R.M., Bangsbo J. (2013). Fibre type-specific change in FXYD1 phosphorylation during acute intense exercise in humans. J. Physiol..

[13] Rasmussen M., Kristensen M., Juel C. (2008). Exercise-induced regulation of phospholemman (FXYD1) in rat skeletal muscle: Implications for Na^+^/K^+^-ATPase activity. Acta Physiol..

[14] Kutz L.C., Mukherji S.T., Wang X., Bryant A., Larre I., Heiny J.A., Lingrel J.B., Pierre S.V., Xie Z. (2018). Isoform-specific role of Na/K-ATPase α1 in skeletal muscle. Am. J. Physiol.-Endocrinol. Metab..

[15] Lubarski I., Karlish S.J., Garty H. (2007). Structural and functional interactions between FXYD5 and the Na^+^-K^+^-ATPase. Am. J. Physiol. Renal. Physiol..

[16] Lubarski I., Pihakaski-Maunsbach K., Karlish S.J., Maunsbach A.B., Garty H. (2005). Interaction with the Na,K-ATPase and tissue distribution of FXYD5 (related to ion channel). J. Biol. Chem..

[17] Jan V., Miš K., Nikolic N., Dolinar K., Petrič M., Bone A., Thoresen G.H., Rustan A.C., Marš T., Chibalin A.V. (2021). Effect of differentiation, de novo innervation, and electrical pulse stimulation on mRNA and protein expression of Na^+^, K^+^-ATPase, FXYD1, and FXYD5 in cultured human skeletal muscle cells. PLoS ONE.

[18] Boon H., Kostovski E., Pirkmajer S., Song M., Lubarski I., Iversen P.O., Hjeltnes N., Widegren U., Chibalin A.V. (2012). Influence of chronic and acute spinal cord injury on skeletal muscle Na^+^-K^+^-ATPase and phospholemman expression in humans. Am. J. Physiol.-Endocrinol. Metab..

[19] Thomassen M., Christensen P.M., Gunnarsson T.P., Nybo L., Bangsbo J. (2010). Effect of 2-wk intensified training and inactivity on muscle Na^+^-K^+^ pump expression, phospholemman (FXYDI) phosphorylation, and performance in soccer players. J. Appl. Physiol..

[20] Thomassen M., Gunnarsson T.P., Christensen P.M., Pavlovic D., Shattock M.J., Bangsbo J. (2016). Intensive training and reduced volume increases muscle FXYD1 expression and phosphorylation at rest and during exercise in athletes. Am. J. Physiol.-Regul. Integr. Comp. Physiol..

[21] Deshmukh A., Steenberg D., Hostrup M., Birk J., Larsen J., Santos A., Kjøbsted R., Hingst J., Schéele C., Murgia M. (2021). Deep muscle-proteomic analysis of freeze-dried human muscle biopsies reveals fiber type-specific adaptations to exercise training. Nat. Commun..

[22] Hostrup M., Bangsbo J. (2017). Limitations in intense exercise performance of athletes–effect of speed endurance training on ion handling and fatigue development. J. Physiol..

[23] Wyckelsma V.L., McKenna M.J., Levinger I., Petersen A.C., Lamboley C.R., Murphy R.M. (2016). Cell specific differences in the protein abundances of GAPDH and Na^+^, K^+^-ATPase in skeletal muscle from aged individuals. Exp. Gerontol..

[24] Wyckelsma V.L., McKenna M.J., Serpiello F.R., Lamboley C.R., Aughey R.J., Stepto N.K., Bishop D.J., Murphy R.M. (2015). Single-fiber expression and fiber-specific adaptability to short-term intense exercise training of Na^+^-K^+^-ATPase α-and β-isoforms in human skeletal muscle. J. Appl. Physiol..

[25] Murphy K.T., Aughey R., Petersen A., Clark S.A., Goodman C., Hawley J.A., Cameron-Smith D., Snow R.J., McKenna M. (2007). Effects of endurance training status and sex differences on Na^+^, K+-pump mRNA expression, content and maximal activity in human skeletal muscle. Acta Physiol..

[26] Lubarski I., Asher C., Garty H. (2011). FXYD5 (dysadherin) regulates the paracellular permeability in cultured kidney collecting duct cells. Am. J. Physiol.-Ren. Physiol..

[27] Fiorenza M., Hostrup M., Gunnarsson T.P., Shirai Y., Schena F., Iaia F.M., Bangsbo J. (2019). Neuromuscular fatigue and metabolism during high-intensity intermittent exercise. Med. Sci. Sports Exerc..

[28] Gunnarsson T.P., Brandt N., Fiorenza M., Hostrup M., Pilegaard H., Bangsbo J. (2019). Inclusion of sprints in moderate intensity continuous training leads to muscle oxidative adaptations in trained individuals. Physiol. Rep..

[29] Fiorenza M., Gunnarsson T., Hostrup M., Iaia F., Schena F., Pilegaard H., Bangsbo J. (2018). Metabolic stress-dependent regulation of the mitochondrial biogenic molecular response to high-intensity exercise in human skeletal muscle. J. Physiol..

[30] Brandt N., Gunnarsson T.P., Hostrup M., Tybirk J., Nybo L., Pilegaard H., Bangsbo J. (2016). Impact of adrenaline and metabolic stress on exercise-induced intracellular signaling and PGC-1α mRNA response in human skeletal muscle. Physiol. Rep..

[31] Nikolić N., Görgens S., Thoresen G., Aas V., Eckel J., Eckardt K. (2017). Electrical pulse stimulation of cultured skeletal muscle cells as a model for in vitro exercise–possibilities and limitations. Acta Physiol..

[32] Hostrup M., Kalsen A., Onslev J., Jessen S., Haase C., Habib S., Ørtenblad N., Backer V., Bangsbo J. (2015). Mechanisms underlying enhancements in muscle force and power output during maximal cycle ergometer exercise induced by chronic β2-adrenergic stimulation in men. J. Appl. Physiol..

[33] Hostrup M., Jessen S., Onslev J., Clausen T., Porsbjerg C. (2017). Two-week inhalation of budesonide increases muscle Na, K ATPase content but not endurance in response to terbutaline in men. Scand. J. Med. Sci. Sport..

[34] Verrey F., Summa V., Heitzmann D., Mordasini D., Vandewalle A., Féraille E., Zecevic M. (2003). Short-term aldosterone action on Na, K-ATPase surface expression: Role of aldosterone-induced SGK1?. Ann. N. Y. Acad. Sci..

[35] Christiansen D., Bishop D.J., Broatch J.R., Bangsbo J., McKenna M.J., Murphy R.M. (2018). Cold-water immersion after training sessions: Effects on fiber type-specific adaptations in muscle K^+^ transport proteins to sprint-interval training in men. J. Appl. Physiol..

[36] Nielsen J.J., Mohr M., Klarskov C., Kristensen M., Krustrup P., Juel C., Bangsbo J. (2004). Effects of high-intensity intermittent training on potassium kinetics and performance in human skeletal muscle. J. Physiol..

[37] Mohr M., Krustrup P., Nielsen J.J., Nybo L., Rasmussen M.K., Juel C., Bangsbo J. (2007). Effect of two different intense training regimens on skeletal muscle ion transport proteins and fatigue development. Am. J. Physiol.-Regul. Integr. Comp. Physiol..

[38] Lemminger A.K., Fiorenza M., Eibye K., Bangsbo J., Hostrup M. (2022). High-Intensity Exercise Training Alters the Effect of N-Acetylcysteine on Exercise-Related Muscle Ionic Shifts in Men. Antioxidants.

[39] Hostrup M., Gunnarsson T.P., Fiorenza M., Mørch K., Onslev J., Pedersen K.M., Bangsbo J. (2019). In-season adaptations to intense intermittent training and sprint interval training in sub-elite football players. Scand. J. Med. Sci. Sport..

[40] Vorup J., Tybirk J., Gunnarsson T.P., Ravnholt T., Dalsgaard S., Bangsbo J. (2016). Effect of speed endurance and strength training on performance, running economy and muscular adaptations in endurance-trained runners. Eur. J. Appl. Physiol..

[41] Gunnarsson T.P., Christensen P.M., Thomassen M., Nielsen L.R., Bangsbo J. (2013). Effect of intensified training on muscle ion kinetics, fatigue development, and repeated short-term performance in endurance-trained cyclists. Am. J. Physiol.-Regul. Integr. Comp. Physiol..

[42] Gunnarsson T.P., Christensen P.M., Holse K., Christiansen D., Bangsbo J. (2012). Effect of additional speed endurance training on performance and muscle adaptations. Med. Sci. Sports Exerc..

[43] Wyckelsma V.L., Levinger I., Murphy R.M., Petersen A.C., Perry B.D., Hedges C.P., Anderson M.J., McKenna M.J. (2017). Intense interval training in healthy older adults increases skeletal muscle [3H] ouabain-binding site content and elevates Na^+^, K^+^-ATPase α2 isoform abundance in Type II fibers. Physiol. Rep..

[44] Perry B.D., Wyckelsma V.L., Murphy R.M., Steward C.H., Anderson M., Levinger I., Petersen A.C., McKenna M.J. (2016). Dissociation between short-term unloading and resistance training effects on skeletal muscle Na^+^, K^+^-ATPase, muscle function, and fatigue in humans. J. Appl. Physiol..

[45] Christiansen D., Eibye K.H., Rasmussen V., Voldbye H.M., Thomassen M., Nyberg M., Gunnarsson T.G., Skovgaard C., Lindskrog M.S., Bishop D.J. (2019). Cycling with blood flow restriction improves performance and muscle K^+^ regulation and alters the effect of anti-oxidant infusion in humans. J. Physiol..

[46] Altarawneh M.M., Hanson E.D., Betik A.C., Petersen A.C., Hayes A., McKenna M.J. (2020). Effects of testosterone suppression, hindlimb immobilization, and recovery on [3H] ouabain binding site content and Na^+^, K^+^-ATPase isoforms in rat soleus muscle. J. Appl. Physiol..

[47] Obradovic M., Stanimirovic J., Panic A., Bogdanovic N., Sudar-Milovanovic E., Cenic-Milosevic D., Isenovic E.R. (2017). Regulation of Na^+^/K^+^-ATPase by estradiol and IGF-1 in cardio-metabolic diseases. Curr. Pharm. Des..

[48] Nam J.-S., Hirohashi S., Wakefield L.M. (2007). Dysadherin: A new player in cancer progression. Cancer Lett..

[49] Codella R., Della Guardia L., Terruzzi I., Solini A., Folli F., Varoni E.M., Carrassi A., Luzi L. (2021). Physical activity as a proxy to ameliorate inflammation in patients with type 2 diabetes and periodontal disease at high cardiovascular risk. Nutr. Metab. Cardiovasc. Dis..

[50] Della Guardia L., Carnevale Pellino V., Filipas L., Bonato M., Gallo G., Lovecchio N., Vandoni M., Codella R. (2023). Nordic Walking Improves Cardiometabolic Parameters, Fitness Performance, and Quality of Life in Older Adults With Type 2 Diabetes. Endocr. Pract..

[51] Hostrup M., Lemminger A.K., Stocks B., Gonzalez-Franquesa A., Larsen J.K., Quesada J.P., Thomassen M., Weinert B.T., Bangsbo J., Deshmukh A.S. (2022). High-intensity interval training remodels the proteome and acetylome of human skeletal muscle. Elife.

[52] Hostrup M., Onslev J., Jacobson G.A., Wilson R., Bangsbo J. (2018). Chronic β2-adrenoceptor agonist treatment alters muscle proteome and functional adaptations induced by high intensity training in young men. J. Physiol..

[53] Bergström J. (1975). Percutaneous needle biopsy of skeletal muscle in physiological and clinical research. Scand. J. Clin. Lab. Investig..

[54] Bangsbo J., Krustrup P., Gonzalez-Alonso J., Boushel R., Saltin B. (2000). Muscle oxygen kinetics at onset of intense dynamic exercise in humans. Am. J. Physiol. Regul. Integr. Comp. Physiol..

[55] Nyberg M., Christensen P.M., Mortensen S.P., Hellsten Y., Bangsbo J. (2014). Infusion of ATP increases leg oxygen delivery but not oxygen uptake in the initial phase of intense knee-extensor exercise in humans. Exp. Physiol..

[56] Lindinger M.I., Spriet L.L., Hultman E., Putman T., McKelvie R.S., Lands L.C., Jones N.L., Heigenhauser G.J. (1994). Plasma volume and ion regulation during exercise after low- and high-carbohydrate diets. Am. J. Physiol..

[57] Putman C.T., Jones N.L., Heigenhauser G.J. (2003). Effects of short-term training on plasma acid-base balance during incremental exercise in man. J. Physiol..

[58] Skovgaard C., Christiansen D., Christensen P.M., Almquist N.W., Thomassen M., Bangsbo J. (2018). Effect of speed endurance training and reduced training volume on running economy and single muscle fiber adaptations in trained runners. Physiol. Rep..

[59] Thomassen M., Hostrup M., Murphy R.M., Cromer B.A., Skovgaard C., Gunnarsson T.P., Christensen P.M., Bangsbo J. (2018). Abundance of ClC-1 chloride channel in human skeletal muscle: Fiber type specific differences and effect of training. J. Appl. Physiol..

[60] Ino Y., Gotoh M., Sakamoto M., Tsukagoshi K., Hirohashi S. (2002). Dysadherin, a cancer-associated cell membrane glycoprotein, down-regulates E-cadherin and promotes metastasis. Proc. Natl. Acad. Sci. USA.

[61] Tsuiji H., Takasaki S., Sakamoto M., Irimura T., Hirohashi S. (2003). Aberrant O-glycosylation inhibits stable expression of dysadherin, a carcinoma-associated antigen, and facilitates cell-cell adhesion. Glycobiology.

[62] Hotta T., Nariai Y., Kajitani N., Kadota K., Maruyama R., Tajima Y., Isobe T., Kamino H., Urano T. (2023). Generation of the novel anti-FXYD5 monoclonal antibody and its application to the diagnosis of pancreatic and lung cancer. Biochimie.

[63] Tokhtaeva E., Sun H., Deiss-Yehiely N., Wen Y., Soni P.N., Gabrielli N.M., Marcus E.A., Ridge K.M., Sachs G., Vazquez-Levin M. (2016). The O-glycosylated ectodomain of FXYD5 impairs adhesion by disrupting cell–cell trans-dimerization of Na, K-ATPase β1 subunits. J. Cell Sci..

